# Examining the Contrast Avoidance Model to Understand the Association between Generalized Anxiety Disorder and Sleep Difficulties

**DOI:** 10.1007/s11126-025-10160-8

**Published:** 2025-06-05

**Authors:** Whitney S. Shepherd, Mary J. Schadegg, Laura J. Dixon

**Affiliations:** 1 Department of Clinical Child Psychology, University of Kansas, Lawrence, KS USA; 2 Department of Psychiatry, NYU Grossman School of Medicine, NYU Langone Health, New York, NY USA; 3 Department of Psychology, University of Mississippi, University, Oxford, MS USA

**Keywords:** Generalized anxiety disorder, Sleep difficulties, Insomnia, Contrast avoidance model, Emotional avoidance

## Abstract

Generalized anxiety disorder (GAD) features excessive worry, paired with physiological arousal that causes distress and interferes with daily activities. Individuals with GAD symptoms often experience persistent sleep problems, which may worsen clinical outcomes. The Contrast Avoidance Model (CAM; Newman & Llera, 2011) posits that worry is used in GAD to avoid emotional shifts by perpetuating a negative state. Research has demonstrated the CAM uniquely explains GAD pathology and may similarly explain co-occurring sleep difficulties. To better understand the high rates of sleep difficulties in GAD symptomology, this study examined the role of contrast avoidance in this relationship in a sample of individuals with anxiety symptoms. Greater contrast avoidance was expected to be positively correlated with sleep difficulties, and GAD severity was expected to be indirectly associated with sleep difficulties via greater engagement in contrast avoidance. A sample of 255 anxious adults was recruited online, and participants completed self-report measures of GAD severity, contrast avoidance, and sleep difficulties. Significant correlations were observed between study variables. The mediation model accounted for 28% of the variance in sleep difficulties and revealed a significant indirect effect of contrast avoidance in the association between GAD severity and sleep difficulties. These results indicate that contrast avoidance may be important when examining sleep difficulties among those with GAD symptoms.

## Introduction

Approximately 6.8 million adults in the United States are diagnosed with generalized anxiety disorder (GAD) annually (Anxiety & Depression Association of America [ADAA], 2021 [3]). GAD features excessive worry and anxiety about multiple domains (e.g., family, health, finances; [23, 63]) paired with physiological symptoms (e.g., restlessness, irritability, American Psychiatric Association [APA], 2013 [2]). Theoretical perspectives purport that individuals with GAD use worry to manage distress [8], avoid unpleasant somatic arousal (e.g., increased heart rate [10, 12],), and prepare for feared events [11]. This worry disrupts daily activities [8, 25] and is associated with problems in physical health, relationships, and workplace performance [38, 56, 93].

One significant negative consequence of chronic worry in those with GAD is sleep difficulties. Sleep difficulties (i.e., difficulty falling asleep, staying asleep, restless sleep), with insomnia being the most common sleep issue, affect 16 to 43.6% of individuals with GAD symptoms [19, 73]. Sleep problems have been associated with a myriad of adverse outcomes, including worse quality of life [88], suicidality [26], and increased health risk [47]. Research has shown that anxiety is significantly associated with sleep difficulties and that individuals with insomnia experience significantly higher presleep arousal and associated worry than individuals without insomnia [27, 61]. Indeed, those with GAD and sleep difficulties have been shown to experience significantly worse health-related outcomes, increased impairment, and worse anxiety treatment responses than those without sleep issues [77, 78]. Prior evidence suggests that general worry predicts insomnia symptom severity and that sleep-specific worry is associated with poor sleep quality such that worries regarding poor sleep apprehension lead to worsened sleep outcomes [74]. Using an hourly diary methodology and an electrocardiogram device, researchers found that prolonged worry significantly predicts high heart rate and low heart rate variability during nocturnal sleep periods [16], causing sleep disruption. Further, disturbances in sleep have been shown to predict higher levels of worry as well, particularly among those with GAD [21, 24, 57, 89]. This evidence suggests there may be a reciprocal relationship, wherein excessive worry results in disrupted sleep patterns and a poor night of sleep results in additional worries the proceeding day, thereby perpetuating a cycle of sleep difficulties.

The Contrast Avoidance Model (CAM) may help explain sleep difficulties as a result of dysfunctional pattern of coping within GAD. Initially conceptualized within the framework of experiential avoidance [66], the CAM posits that worry functions specifically to avoid sharp *contrasts* in negative emotion, rather than avoidance of the general experience of aversive internal states as theorized by other models of worry (e.g., [8, 9]). That is, individuals with GAD prefer to experience a heightened and constant, negative emotional state and are particularly sensitive to abrupt shifts from neutral or positive states to distressing ones, which can feel more threatening, out of control, and difficult to manage [14, 50, 66]. To minimize this contrast, they seek to maintain a sustained state of negative emotion through chronic worry, thereby reducing the intensity of emotional “spikes” when faced with feared outcomes [50]. In this way, worry serves as a paradoxical form of experiential avoidance, wherein individuals do not avoid emotion entirely, but rather avoid the internal experience of emotional *change*. Importantly, the CAM also highlights that individuals with GAD seek positive emotional contrasts because these shifts feel rewarding (e.g., pleasant surprise of a feared outcome not happening). As such, individuals not only engage in worry to avoid negative contrasts, but also to increase the likelihood of experiencing greater positive emotional contrasts. However, prolonged positive emotional states begin to feel uncomfortable, as individuals feel vulnerable to negative contrasts [50, 52], and consequently, they may return to worry to preemptively dampen positive emotion and protect against future distress [54]. In fact, one study using a college sample found that dampening positive affective states uniquely and strongly predicted GAD symptoms. Another study found that, when compared to a non-anxious sample, individuals with GAD reported significantly greater discomfort with sustaining positive affect, endorsing items such as “I was focusing on the negative, because allowing myself to feel happy leaves me vulnerable to feeling terrible in the end” [5]. Thus, worry is both negatively reinforced (i.e., by reducing emotional spikes) and positively reinforced (i.e., by generating temporary moments of relief), contributing to its persistence over time. Research has found that contrast avoidance behavior significantly predicts GAD severity, above and beyond other well-tested GAD predictors such as intolerance of uncertainty [51]. Further, researchers have demonstrated that individuals with GAD endorsed significantly higher rates of contrast avoidance behavior compared to those without GAD [5].

The CAM may help understand sleep difficulties, as the steady baseline of heightened negative emotionality that is maintained by worry, perpetuates physiological arousal and emotional distress [1] [87], which may lead to poor sleep quality [16, 35, 68]. Indeed, the literature indicates sleep cognitions about the consequences of sleep loss (e.g., worrying about not getting enough sleep, daytime somnolence) trigger a state of autonomic arousal that disturbs sleep [35, 53, 92]. Research has also shown worrying before bed prevents or delays sleep onset [8, 34, 58, 86] and predicts poor sleep quality overall [17]. In considering contrast avoidance tendencies and sleep, sleep-oriented worry may function to increase the likelihood of experiencing greater positive emotional contrasts if adequate sleep is achieved. Yet, at the same time, the state of worry may also be used to stave off the distressing internal experience of feeling a downward shift in their emotions, ultimately resulting in higher levels of arousal and worse sleep outcomes. Consistent with evidence that individuals with GAD feel uncomfortable in positive and neutral states [50, 52], research has also shown individuals with GAD are prone to experiencing relaxation-induced anxiety [43, 65], a phenomenon in which attempts to relax paradoxically lead to spikes in anxiety [15, 37]. In the context of the CAM, attempts to relax may trigger a sense of vulnerability and urgency to engage in worry to prevent a downward emotional shift. Kim and Newman [43] found that the effect of GAD symptoms on relaxation-induced anxiety was fully explained by negative contrast sensitivity. In other words, GAD symptoms were associated with high sensitivity to negative contrasts, which in turn, contributed to increases in anxiety and worry when attempting to relax. Thus, one theory is that bedtime, a period inherently marked by relaxation, may trigger individuals with GAD to engage in sustained worry in order to protect from sudden, drastic spikes in negative emotions, which ultimately leads to arousal, and consequently, poor sleep. However, no studies have directly examined contrast avoidance behaviors and sleep difficulties.

Examining contrast avoidance behaviors is critical for informing the cognitive-behavioral models of GAD and better understanding sleep dysfunctions in those with anxiety pathology. Prior research has linked GAD symptoms [21, 24, 46, 59, 83, 89] and worry [30, 39, 58] to sleep difficulties. Furthermore, the CAM has been implicated in relaxation-induced anxiety [43]—an experience common among individuals with GAD [43, 65]—which may drive increased worry during relaxation attempts at bedtime, ultimately contributing to heightened arousal (Adamis et al., 2024; [87]) and disrupted sleep [16, 17, 35, 53, 68, 92]. Given the prevalence and impact of sleep difficulties in those with GAD [19, 77, 78], such knowledge has the potential to enhance our understanding of co-occurring dysfunctions in GAD and develop treatment implications for these issues. Based on prior research [21, 24, 43, 50, 57, 89], it was hypothesized there would be positive correlations between GAD symptoms, contrast avoidance tendencies, and sleep difficulties. Second, it was predicted that GAD symptoms would be indirectly associated with sleep difficulties through contrast avoidance tendencies.

## Material and Methods

### Participants and Procedures

Participants were recruited from a CloudResearch online panel of individuals who responded to health screener checklist and consistently endorsed a diagnosis of anxiety across multiple administrations [48]. Research suggests that online crowdsourcing platforms are valuable for collecting clinically relevant data among a community-based sample in a reliable and cost-efficient way [62, 84]. Participants completed a series of questionnaires, and a data quality check (i.e., attention check questions, completion of questionnaires, identification of response inconsistencies) was completed by study personnel. Participants received compensation of up to $2.00 for completing the survey. The study was approved by the Institutional Review Board of the University of Mississippi in accordance with the Declaration of Helsinki.

A total of 268 participants provided informed consent. Of those participants, 13 were excluded due to incomplete responses. The final sample included 255 participants who endorsed anxiety (70.6% female). The mean age of the sample was 38.33 years (*SD* = 10.86), and participants identified as White (88.2%), African American (6.7%), Asian/Southeast Asian (5.1%), and Hispanic/Latino (6.3%). Most participants reported some college/university education (88.6%) and were employed full-time (58%). Additional demographic features of the sample are described in Table 1.

**Table 1:** Sample Characteristics (*N* = 255)

	% (*n*)
Sex (at birth)	
Female	70.6 (*180*)
Male	29.0 (*74*)
Race/Ethnicity	
African American	6.7 (*17*)
Asian/Southeast Asian	5.1 (*13*)
White	88.2 (*225*)
Hispanic/Latino	6.3 (*16*)
American Indian/Alaska Native	0 (*0*)
Native Hawaiian/Other Pacific Islander	0 (*0*)
Sexual Orientation	
Heterosexual/Straight	76.9 (*196*)
Gay/Lesbian	5.1 (*13*)
Bisexual	17.3 (*44*)
Questioning	0.4 (*1*)
Region of Residence	
Northwest	22.4 (*57*)
Midwest	25.1 (*64*)
South	33.3 (*85*)
West	19.2 (*49*)
Income	
Less than $10,000	4.7 (*12*)
$10,000 to $24,999	15.7 (*40*)
$25,000 to $49,999	30.2 (77)
$50,000 to $74,999	22.0 (*56*)
$75,000 to $99,999	13.3 (*34*)
$100,000 to $149,999	7.8 (*20*)
$150,000 or more	6.3 (*16*)
Marital/Relationship Status	
Single (never married, living alone, divorced, widowed, etc.)	43.1 (*110*)
Living with a partner as if married	18.8 (*48*)
Married but separated	3.9 (*10*)
Married	34.1 (*87*)

### Measures

#### GAD Symptoms

The Generalized Anxiety Disorder Questionnaire IV (GAD-Q-IV) is a 9-item self-report diagnostic measure used to assess for GAD [71]. Participants identify the most frequent topics of worry, respond to yes/no questions regarding their experience with worry and GAD symptoms, and rate distress and interference with daily life on 8-point Likert scales ranging from 0 (*Not at all/No Distress*) to 7 (*Very Severely/Very Severe Distress*). Total scores range from 0 to 13, with a cutoff score of 5.70 which indicates clinical levels of GAD [71], and has demonstrated an 83% sensitivity and 89% specificity in diagnosing GAD [71]. This measure demonstrates strong test–retest reliability, convergent and discriminant validity [71], and has good internal consistency (α = 0.82; [18]). The current study also demonstrated strong internal consistency (α = 0.83).

#### Contrast Avoidance Tendencies

The Contrast Avoidance Questionnaire- General Emotion (CAQ-GE) was used to measure participants’ general beliefs and behaviors regarding emotional contrast avoidance [50]. Three features of the CAM are assessed, including discomfort with emotional shifts (e.g., *When my emotions fluctuate it makes me feel out of control*), creating and perpetuating negative emotion to avoid negative contrasts (e.g., *I would rather feel bad now, because at least I won’t experience an emotional rollercoaster if terrible things happen*), and tendency to allow positive emotional contrast (e.g., *I prefer to have a pessimistic outlook, so that I can be pleasantly surprised if something good happens*, [50]). This questionnaire has 25 items and participants answered using a 5-point Likert scale of 1 *(not at all true*) to 5 *(absolutely true*). This measure has a cutoff score of 44.50 that has been found to distinguish between those reporting GAD symptoms and non-GAD samples with a sensitivity of 89.7 and a specificity of 89.3 [50]. The measure has demonstrated strong psychometric properties with strong validity and test–retest reliability [50], with a Cronbach alpha of α = 0.96 in the current sample.

#### Sleep Difficulties

The Insomnia Severity Index (ISI) is a 7-item self-report measure used to assess the nature, severity, and impact of insomnia [6]. The items of the ISI evaluate severity of sleep onset, sleep maintenance, early morning awakening problems, sleep dissatisfaction, interference of sleep difficulties with daytime functioning, perception of sleep difficulties by others, and distress caused by sleep difficulties. The total score ranges from 0 to 28, and items are scored on a 5-point Likert scale ranging from 0 (*no problem*) to 4 (*very severe problem*. A total score of 0–7 indicates no insomnia, 8–14 indicates sub-threshold insomnia, 15–21 indicates moderate insomnia, and 22–28 indicates severe insomnia; however, a cutoff of 10 was optimal for detecting insomnia cases in a community sample with a sensitivity of 86.1 and a specificity of 87.7 [60]. The ISI has strong reliability and validity [60], and the current sample demonstrated a high internal consistency (α = 0.92).

## Results

### Sample Characteristics and Bivariate Correlations

Means, standard deviations, ranges, and bivariate correlations for GAD severity, contrast avoidance, and sleep difficulties are reported in Table 2. Within the sample, 72.5% of participants reported GAD symptoms at or above the clinical cutoff on the GAD-Q-IV. With regard to sleep, 32.6% of the sample scored at or above the clinical cutoff on the ISI, indicating clinical levels of sleep difficulties. Lastly, 75.8% of participants scored above the clinical cutoff on the CAM-GE. As predicted, there was a positive correlation between GAD symptoms and sleep difficulties (*r* = 0.53; *p* < 0.01) and contrast avoidance (*r* = 0.60; *p* < 0.01), both yielding large effects. Contrast avoidance tendencies were also positively correlated with sleep difficulties, yielding a medium effect (*r* = 0.43; *p* < 0.01).

**Table 2:** Means, Standard Deviations, and Bivariate Correlations of Study Variables

	1	2	3
1. GAD Severity	-		
2. Contrast Avoidance	.60*	-	
3. Sleep Difficulties	.53*	.43*	-
Mean	8.05	64.18	5.95
*SD* Observed Range	3.48 0–13	22.86 25–125	5.32 0–28

GAD severity = scores on the Generalized Anxiety Disorder Questionnaire IV [71], contrast avoidance = Contrast Avoidance Questionnaire [50], sleep difficulties = Insomnia Severity Scale [6]

*p <.01

### Mediation Analysis

**Fig. 1:**
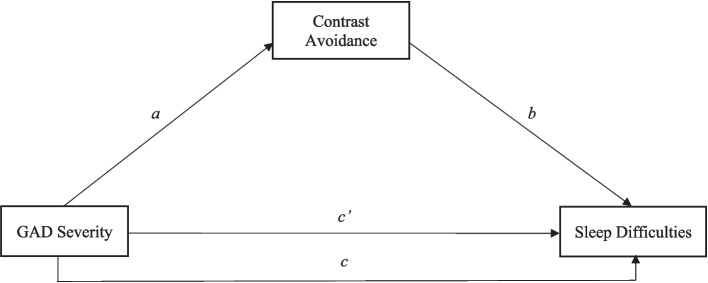
Conceptual diagram of the proposed model testing the direct and indirect effects of GAD severity on sleep difficulties through contrast avoidance. GAD Severity = Generalized Anxiety Disorder Questionnaire IV; contrast avoidance = Contrast Avoidance Questionnaire- General Emotion; sleep difficulties = Insomnia Severity Index

A mediation analysis was conducted using the PROCESS Macro [36] to test the hypothesis that GAD severity would predict elevated sleep difficulties mediated by contrast avoidance tendencies. A bootstrap sampling method was used to generate 5,000 resamples of total, direct, and indirect effects of GAD symptom severity on sleep outcomes, and confidence intervals (CI 95%) were calculated. Results were considered statistically significant if the confidence intervals reported a range that excluded zero. Data assumptions were met, including linearity, normality, multicollinearity, and outliers were examined. In the model, GAD severity was the independent variable, contrast avoidance was the mediator variable, and sleep difficulties was the outcome variable (see Fig. 1). The overall model was significant (*F* = 90.45, *p* <. 001), yielding a large effect size determined by R^2^ [20], with GAD symptom severity accounting for 28% of the variance in sleep difficulties, as well as the direct path between GAD severity and sleep difficulties (*B* = 0.66, *SE* = 0.10; 95% CI [0.46, 0.86]). As predicted, the indirect path between GAD severity and sleep difficulties was significant, (*B* = 0.16, *SE* = 0.07; 95% CI [0.03, 0.29]). See Table 3 for the full results and effect sizes for each path. Lastly, given that the GAD-Q-IV includes an item assessing sleep disturbance, analyses were also conducted with this item omitted, and similar results were observed.

**Table 3:** Mediation Results for GAD Severity on Sleep Difficulties through Contrast Avoidance

Model	*R*^2^	Path	*β*	*B*	*SE*	*t*	*p*	CI* (l)*	CI* (u)*
Sleep difficulties	.282						<.001		
		*a*	0.60	3.91	0.33	11.78	<.001	3.26	4.57
		*b*	0.17	0.04	0.15	2.59	.0102	0.01	0.07
		*c*	0.51	0.81	0.08	9.93	<.001	0.65	0.97
		*c’*	0.42	0.66	0.10	6.52	<.001	0.46	0.86
		a*b	0.10	0.16	0.07			0.03	0.29

*GAD* generalized anxiety disorder. Contrast avoidance = Contrast Avoidance Questionnaire IV; Sleep difficulties = Insomnia Severity Scale. *R*^2^ represents the results for the full mediation model. β = standardized beta coefficients, with 0.10–0.29 = small effect, 0.30–0.49 = medium effect, and ≥ 0.50 = large effect [20, 72]. *B* = unstandardized regression coefficient, *SE* = standard error; CI (*l*) = lower bound of 95% confidence interval; CI (*u*) = upper bound of 95% confidence interval; a*b represents the indirect path

## Discussion

To further understand the high rates and impact of sleep difficulties in GAD, this study evaluated the potential role of contrast avoidance in the association between GAD symptoms and sleep difficulties in an anxious sample. The majority of the current sample endorsed clinical levels of GAD symptoms, and approximately one-third of participants reported clinically significant sleep difficulties, which is consistent with rates observed in anxious samples (e.g., [19]). Most of the sample also scored beyond the clinical cutoff for contrast avoidance, which aligns with previous research demonstrating similar elevations of contrast avoidance behaviors among clinically anxious adults [50, 80]. The prevalence of contrast avoidance behaviors, co-occurring sleep difficulties, and GAD symptoms emphasize the high levels of distress in this sample.

Supporting the first hypothesis, the results demonstrated significant associations between GAD severity, contrast avoidance, and sleep difficulties. Consistent with prior research [33], the results indicate that GAD severity is associated with sleep difficulties. Additionally, GAD was associated with contrast avoidance, which aligns with research indicating that avoidance of shifts in negative emotions is common among individuals with GAD [7, 22, 49, 64, 66, 70], and associated with GAD symptom severity [55]. The current results extend previous research by establishing a positive correlation between contrast avoidance tendencies and sleep difficulties.

The second hypothesis predicting that contrast avoidance would function as a mediator in the relationship between GAD symptom severity and sleep difficulties was supported. As expected, GAD symptom severity was indirectly associated with sleep difficulties via contrast avoidance. That is, these findings suggest that individuals with elevated GAD symptoms may have strong tendencies to avoid emotional contrasts, which in turn may worsen sleep difficulties. Given findings that relaxation-induced anxiety is a common experience among individuals with GAD [43], individuals with GAD may feel vulnerable to negative contrasts during the critical pre-bedtime period when gradual relaxation typically occurs, which may prompt increased anxiety and arousal. These findings lend support to prior findings indicating that the deliberate increase in negative emotions and worry produces physiological arousal [1] [5], thereby undermining sleep quality [91]. Additional research is needed to replicate these findings in experimental and longitudinal studies to support the directionality of these findings,however, if supported, these findings suggest that individuals with GAD may experience exacerbated sleep problems by consistently engaging in maladaptive emotional management strategies, such as avoidance of shifting emotional states. Over time, the temporary relief yielded by avoiding emotional shifts by engaging in worry may perpetuate the use of poor coping skills and inhibit one’s ability to effectively respond to anxiety. Thus, it may be helpful to integrate skills in interventions to improve the regulation of emotions for individuals who avoid shifts in their negative emotions. Ultimately, these findings provide valuable information to the GAD literature by helping to understand what emotional processes may be related to negative sleep outcomes among individuals with GAD.

Although the current study provides a valuable contribution to the literature, several limitations should be considered. First, this study relied on online, self-report questionnaires, which may be prone to bias (i.e., self-report bias, social desirability; [45, 82]). Future studies could expand upon these findings by incorporating multiple modes of measurement, such as the use of structured diagnostic interviews and sleep assessments. Second, the sample included community members who reported elevated anxiety symptoms. Additional research is needed to understand if the current findings generalize to clinical and treatment-seeking populations. Third, this study was a cross-sectional design,therefore, causality cannot be inferred, and temporal mediation cannot be determined. However, the results provide preliminary support for considering contrast avoidance as a potential mechanism in future studies. An important next step is to extend this work by evaluating changes in GAD symptoms, sleep difficulties, and contrast avoidance through longitudinal study designs and experimental inductions (e.g., [49]). Lastly, the sample was primarily comprised of White women. Although research suggests that a GAD diagnosis is most common in this demographic [4, 40], it is important to examine these constructs in minority populations due to the increased severity of anxiety symptoms seen in these groups [90].

In sum, the current findings enhance the understanding of sleep difficulties among individuals with GAD and provide further insight into how researchers may develop effective treatment for these dysfunctions. Although research has investigated contrast avoidance in relation to other domains of impairment (e.g., interpersonal issues, problem-solving difficulties; [29, 51]) and GAD [14], no studies have examined the relationship between contrast avoidance and sleep difficulties. Additional research is needed to further investigate contrast avoidance as a function of maladaptive coping [66], however, if supported, sleep difficulties may be alleviated by targeting contrast avoidance behaviors among individuals with GAD. Although little research has examined contrast avoidance tendencies as a specific treatment target, studies have identified the potential benefit of focusing on contrast avoidance [66, 69] through exposure to negative contrasts [64, 69, 70] and savoring positive emotions [44]. In addition, applied relaxation, which refers to deliberate strategies to quickly induce relaxed states and counteract anxiety responses [75], has shown to be an effective component of cognitive-behavioral therapy to treat GAD [13,76,85]. Given that applied relaxation may counterintuitively induce anxiety in individuals with heightened sensitivity to downward emotional shifts, this treatment technique may be most effective for decreasing contrast avoidance sensitivity when combined with exposure to negative contrasts [31,79]. In effect, this combined approach would be structured by first teaching relaxation skills, mindfulness, and acceptance of aversive internal experiences (i.e., emotional contrasts, followed by exposures to negative emotional contrasts via a feared outcome. This approach would allow the individual to experience the full range of an emotional increase from a relaxed state to a negative affective and aroused state [79]. Additionally, similar to the first approach, mindfulness training, which focuses on increasing awareness of internal experiences and overall disrupting cycles of worry, has found to be effective in reducing worry and consequently, improving sleep outcomes Gao et al., [32]. Although further research is needed to assess the effectiveness of combining relaxation and mindfulness with an exposure component, these preliminary findings suggest that perhaps improving emotional contrast avoidance, in turn, improves sleep difficulties overall, thus, highlighting the Contrast Avoidance Model as an important GAD framework in which treatment can be developed upon.

## Data Availability

The data that support the findings of this study may be available upon request to the corresponding author. Data are not publicly available due to privacy and ethical restrictions.
